# Old people’s preference for nursing homes in East China: a discrete choice experiment

**DOI:** 10.1186/s12912-024-01907-y

**Published:** 2024-04-22

**Authors:** Yaqin Zhong, Xiaojun Guo, Yitong Liu, Yaning Wang, Yanan Wang, Yan Song, Rujian Lu

**Affiliations:** 1 School of Public Health, Nantong University, Nantong, China; 2 School of Science, Nantong University, Nantong, China; 3 School of Nursing, Nantong University, Nantong, China; 4 Third Affiliated Hospital of Nantong University, Nantong, China; 5 Nantong Third People’s Hospital, Nantong, China

**Keywords:** Nursing homes, Preferences, Discrete choice experiment

## Abstract

**Background:**

The aged people who live in nursing home are predicted to keep growing in the following decades. There are both quantitative imbalance and structural imbalance in the utilization of nursing homes in China. This study aimed to analyze old people’s preference for nursing homes and help the government optimize resource allocation.

**Methods:**

A discrete choice experiment (DCE) was conducted and six attributes of nursing homes including monthly fee, distance from home, geographical location, medical facilities, environment of nursing homes and nursing staff were determined. Respondents were recruited from Nantong and Yangzhou city, China. In each city, two communities or villages were randomly selected. In each community/village, about 65 old people were randomly selected. Analysis was conducted using mixed logit regression models to determine preferences for potential attributes.

**Results:**

A total of 233 old people were included in the analysis. The findings indicated that all six attributes were statistically significant factors for participants. “Professional nursing staff” was the most important characteristic to participants, followed by “Medical facilities”. Compared with female, the males preferred professional nursing staff (β = 2.939 vs. β = 2.643, *P* < 0.001), medical facilities (β = 1.890 vs. β = 1.498, *P* < 0.001), and the environment (β = 0.752, *P* < 0.01). For different age groups, participants aged 60–69 didn’t pay attention to distance and location, while those aged 80 and above only paid attention to professional nursing staff and medical facilities.

**Conclusions:**

The present study provides important insights into the characteristics of nursing home that are most preferred by old people. Authorities should take into account old people’s preference in the planning, design and evaluation of nursing homes.

**Supplementary Information:**

The online version contains supplementary material available at 10.1186/s12912-024-01907-y.

## Background

China has the world’s largest aging population and is facing unprecedented challenges in population aging. According to China’s seventh census, the proportion of people aged 65 and above among the total population was about 13.8% in 2020 and is predicted to reach about 30% by 2050 [1]. The change of the age structure, combined with the country’s rapid development, have had significant influence on the traditional family support system.

Because of the Chinese “one child per family” policy, which was introduced in 1979, Chinese family structure has been changed from the traditional stem families to nuclear families [2]. Though this policy was revoked in 2015, the adjustment is unlikely to slow down the pace of population ageing. For an adult couple, it is extremely difficult to provide support for their combined four aging parents while taking care of their own child. The care function provided by family members for their old generations inevitably weakened. In addition, due to rapid urbanization and increased labor mobility, caregiving by relatives has become strained [3, 4].

Though a body of researches indicated most old people prefer living and being cared in their own homes, the great importance of residential care facilities cannot be neglected [5, 6]. However, though elderly care facilities continued to develop, the supply does not match the demand well. According to Statistical Bulletin on the Development of Civil Affairs in China, 2022, the vacancy rate of elderly care institutions in China stands about 50% [7]. A study conducted in Beijing, China, showed that the bed vacancy rate of nursing home is about 47% [8]. More significantly, a notable phenomenon has emerged that numerous old people had to endure waiting periods ranging from months to even a year for a nursing bed at downtown care facilities, while simultaneously many nursing beds at suburban care facilities remain vacant [5]. So there are both quantitative imbalance and structural imbalance in the utilization of suburban and downtown care facilities [5].

There are enormous variations among Chinese older people in terms of needs, attitude and affordability toward nursing homes [9–11]. Since the development of nursing home is largely based on top-down planning, the provision of nursing home may not always reflect the preference of old people. The mismatch between supply and preferences has been one of the main reasons for under-utilization of nursing homes, which leads to waste of public resources.

The preference for nursing homes is defined as the expectation and choice of the old people, and it represents an individual desire, hopes and expectations of old people for their life in their late years [12]. The aged people who live in nursing home are predicted to keep growing in the following decades. Wei etc. found that the preference for long term care facilities among old people is affected by a range of factors, including age, education level, income, residence, and self-care ability [9]. Wang [13] found the willingness of the elderly to enter the nursing institution is influenced by individual, family environment and community environment, including age, living with spouse and children, house ownership and availability of home healthcare services. Yang found living alone, medical payment, mental illness were the influencing factors of the disabled elderly to receive institutional care [14]. A study conducted in 7 cities in China indicated that intergenerational family support is a significant driver on the willingness of the old people to choose nursing care. The females who are younger, get higher education, have higher income, have medical insurance are more willing to choose nursing care [15]. Du found that compared with home nursing, long-lived old people are more inclined to support professional institutions than younger people. With the increase of age, the physical fitness of the old people will decline, and professional nursing homes can manage their daily life [12].

Some existing studies also pay attention to different attributes of nursing homes. Jingyi, M found that the light condition is more important for the elderly with poor vision, and the bad sound environment will affect their mood and health [16]. Richard provided evidence that quality and changing care needs are among the factors in transfers between nursing homes. Lower quality of care increase the likelihood of referral [17]. Rachel etc. conducted discrete selection experiments with nursing home residents or family member agents. The results showed that feeling at home in the residents’ own room was the most important characteristic [18]. Pablo pointed out that nursing homes and beds tend to be concentrated in areas with high demand and are more prevalent in cities with aging populations and higher incomes. Long-term care services, especially nursing homes, tend to cluster in urban and central areas [19].

In general, the existed studies have been predominantly focused on need and influencing factors for old age care, there are few evidence regarding preferences for various attributes of nursing homes in mainland China. Most studies used logistic regression [12, 20, 21]. The utilization of the nursing home is a response to the degree that the demand is met. It is important to explore how the different attributes of nursing homes are valued by the potential aged customers. Therefore, this study aimed to bridge research gaps by assess the preference for nursing homes of old people, and to better understand what characteristics of the nursing home they preferred. The internationally advanced preference analysis method, discrete choice experiment, was adopted to measure the preference of the old people, so as to make up for the shortcomings of traditional research methods. The present study provides countermeasures and suggestions for promoting the construction and development of nursing homes, and provides data support and decision-making reference for providing accurate elderly care services.

## Methods

### Study design

A cross-sectional design was employed in the present study. A discrete choice experiment (DCE) was used to explore the choice preference of old people for nursing homes. The DCE involved choices between two hypothetical scenarios of nursing homes, described by attributes and levels. The respondents were asked to choose one of them according to their preference.

### Study population

Followed the rule of thumb proposed by Johnson and Orme [23], we calculated the sample size of the present study.

$$\mathrm N>500\mathrm c/\mathrm a\ast\mathrm b$$

Where N is the minimum sample size of the study, *c* is the maximum number of levels in the attribute, *a* is the number of choice tasks in DCE and *b* is the number of profiles in each choice task. Thus, the sample size of the present should be N > 500*3/9*2, at least 83 respondents.

**Fig. 1:**
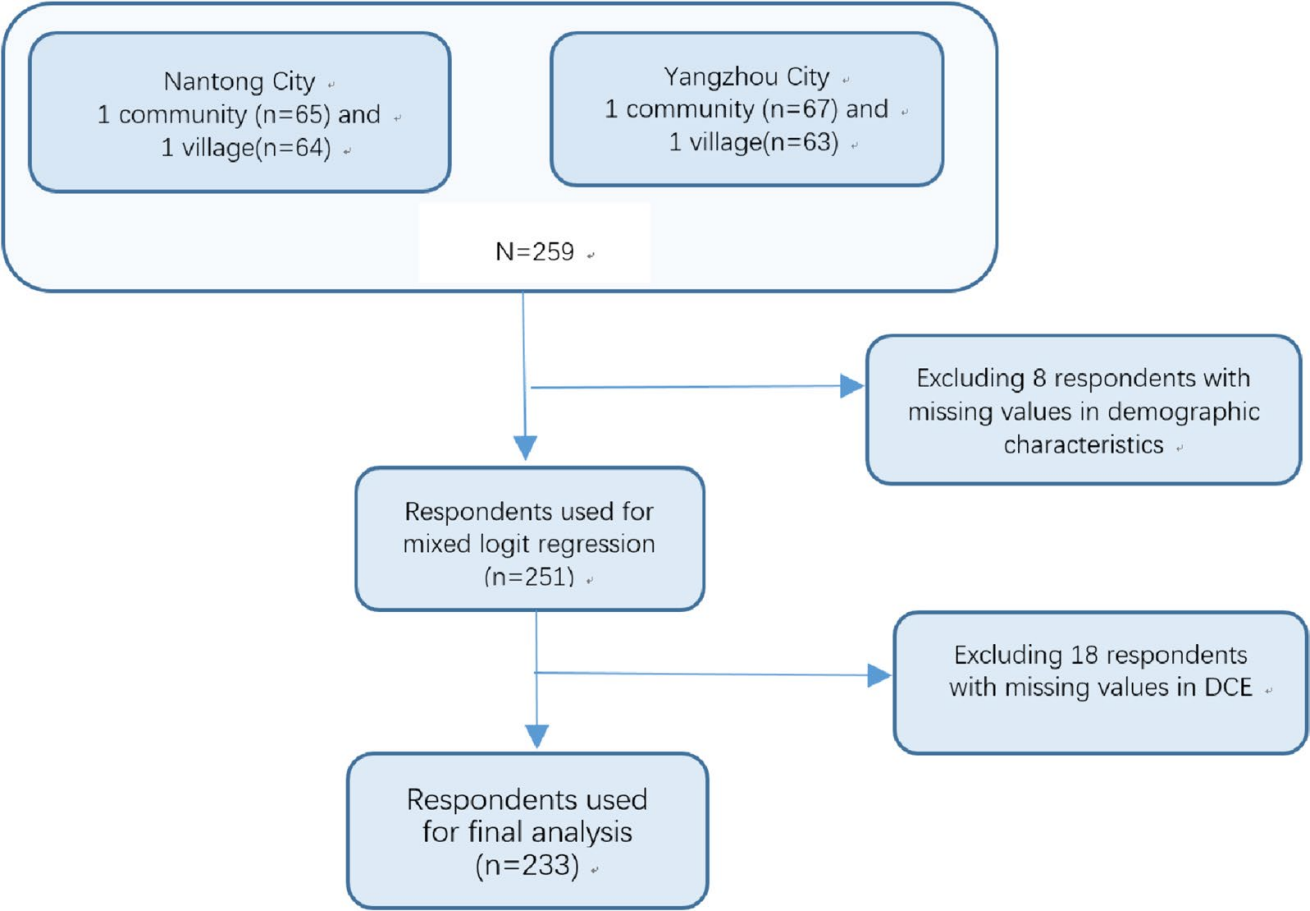
The flow chart of the sample

Respondents were recruited from Nantong and Yangzhou city. These two cities were located in Jiangsu province, China. In 2022, these two areas had entered the severely aging society. In each city, two communities or villages were randomly selected. In each community/village, about 65 old people were randomly selected. The inclusion criteria of the sample included: 1) local residents aged 60 years and above; 2) normal cognitive function; 3) agree to participant in the survey. A total of 259 respondents were invited to participate in the survey and finally 233 valid questionnaires were collected (Fig. 1). The response rate was about 90.0%.

### Identification of DCE attributes and level

Research guideline recommendation of DCE were followed and qualitative and quantitative approaches were combined to develop attributes and levels [22, 23]. In DCE tasks, respondents were presented with certain “profiles scenario” consisting of “attributes” and were asked to evaluate them.

The attributes and levels of the DCE were based on literature and in-depth qualitative interview (see Supplementary file). Six attributes were identified including monthly fee, distance, location, medical facility, environment of the nursing homes and nursing staff. Attributes and corresponding levels were shown in Table 1.

**Table 1:** Attributes and levels for DCE choice questions

Attributes	Definition	Levels	Code
Monthly fee	Monthly care charge for nursing homes	≤CNY2000	0
		CNY2001-4000	1
		>CNY4000	2
Distance	Distance from home to nursing homes by vehicle	≤30minutes	0
		31-60 minutes	1
		>60 minutes	2
Location	Location of the nursing homes	downtown	1
		suburb	0
Medical facility	Whether the nursing home is equipped with medical equipment	yes	1
		no	0
Environment	Environment of the nursing home	good	1
		fair	0
Nursing staff	Whether the nursing staff is professional	professional	1
		not professional	0

- “Monthly fee” was defined as monthly care charge for nursing homes. It is differences as “CNY 2000”, “CNY 2001–4000”, “CNY 4000”, according to old people’s income and the general level of nursing home expense.
- “Distance” is the time distance measured by vehicle from the respondents’ home to the nursing homes. Three levels are set for it as “30 minutes”, “31–60 minutes” and “60 minutes”.
- “Location” means where the nursing home are located, including two levels of “downtown” and “suburb”.
- “Medical facility” is whether the nursing home is equipped with medical equipment.
- “Environment” indicates the environment of the nursing home, including two levels of “good” and “fair”.
- “Nursing staff” is the main concerned factor of the old people for the nursing home in our in-depth review. It is directly related to the quality of service in the nursing home because professional nursing staff can provide professional services.

### Questionnaire design and quality control

The survey was structured as follows: the description and aims of the research, socio-demographic characters of the respondents, health status and discrete choice questions. Socio-demographic characters include age (60–69, 70–79, >=80), gender (male, female), marriage status (in marriage, others), education (primary school and below, middle school, high school and above), occupation (employed, not employed), monthly income (< 2000, 2000–5000, > 5000) and living arrangement (living alone, not alone). Health status included self-rated health (good, fair, poor) and chronic diseases (yes, no). A total of six attributes were included in DCE. Four attributes contained two levels and two attributes contained three levels. 144 (3^2^ × 2^4^) different scenarios were generated. A D-optimal design was conducted to produce 16 choice tasks. In order to reduce the burden on each participant, 16 choice tasks were split into two blocks. In terms of quality control, a repeated question was added into each block to test the internal validity of the survey. Each participant had to complete 9 tasks. They were asked to make choices between a pair of hypothetical scenarios reflecting the characteristics of two nursing homes. The example of DCE question was provided in Table 2. Additionally, to ensure that respondents can read and understand the scenarios, the survey was conducted by trained investigators from Nantong University.

**Table 2:** An example of discrete choice question

	Nursing home 1	Nursing home 2
Monthly fee	> CNY4000	≤CNY2000
Distance	31 ~ 60 min	> 60 min
Location	downtown	suburb
Medical facility	no	has
Environment	good	fair
Nursing staff	professional	not professional
Which nursing home would you prefer to choose	□	□

### Ethical considerations

The present study was approved by the Ethics Committee of Nantong University (protocol code: 2,021,014). All respondents were assured that the information would only be used for research purpose and informed consent was obtained.

### Data analysis

A random utility theory framework was used to analysis the data from the DCE. Random utility theory assumes that the utility of a choice consists of functions of being observed (such as attributes and levels) and unobserved and the respondents will choose maximize utility when making choices [24]. The utility function can be specified as follows:

1 $$U_{ni}=V_{ni}+\varepsilon_{ni}=\beta x_{ni}+\varepsilon_{ni}$$

*V*_*ni*_ is the observable utility, also known as the fixed utility. The utility of attributes and levels is expressed by *βx*_*ni*_, and *ε*_*ni*_ is the unobserved utility. The utility contained random variables, so the utility of each choice is also random. Thus, the probability function *P*_*ni*_ for decision maker n to choose i can be described as:

2 $$\begin{array}{l}P_{ni}=Prob\left(U_{ni}>U_{nj},\forall j\neq i\right)\\=Prob\left(U_{ni}+\varepsilon_{ni}>V_{nj}+\varepsilon_{ni},\forall j\neq i\right)\\=Prob\left(\varepsilon_{nj}-\varepsilon_{ni}<V_{ni}-V_{nj},\forall j\neq i\right)\end{array}$$

Assume that *ε*_*ni*_ is an Independent and Identically Distributed (IID) random variable and subject to the type I Extreme Value Distribution, the *P*_*ni*_ can be written as:

$$P_{ni}=\frac{\exp\left(\beta_ix_n\right)}{\sum\nolimits_j^J\exp\left(\beta_ix_n\right)}$$

In this study, conditional logit and mixed logit were conducted for regression analysis respectively. The values of Akaike information criterion (AIC) and Bayesian information criterion (BIC) of each model were compared, which is commonly used for model selection in random utility framework.

## Results

### Descriptive statistics

A total of 233 old people completed the DCE questionnaire. The age of the participants between 60 and 69, 70 to 79, 80 and above accounted for 38.2%, 47.2% and 14.6%, respectively. The females accounted for 57.1%. 83.3% of the respondents were in marriage. More than half (62.7%) of them were primary school and below. Most of them were not employed. 15.5% of the participants lived alone and 76.4% of them had chronic diseases. The participants rated their health as good, fair and poor accounted for 58.4%, 25.8% and 15.8%, respectively (Table 3).

**Table 3:** Demographic characteristics of the participants

Characteristics	*N* = 233	
	n	%
Age(y)		
60–69	89	38.2
70–79	110	47.2
≥ 80	34	14.6
Survey district		
Urban	104	44.6
Rural	129	55.4
Gender		
Male	100	42.9
Female	133	57.1
Marriage status		
In marriage	194	83.3
Others	39	16.7
Education		
Primary school and below	146	62.7
Middle school	41	17.6
High school and above	46	19.7
Occupation		
Employed	47	20.2
Not employed	186	79.8
Monthly income (CNY)		
< 2000	143	61.4
2000–5000	72	30.9
> 5000	18	7.7
Living alone		
Yes	36	15.5
No	197	84.5
Chronic disease		
Yes	178	76.4
No	55	23.6
Self-rated health		
Good	136	58.4
Fair	60	25.8
Poor	37	15.8

### Preference for nursing homes of the total sample

In this study, two models of conditional logit and mixed logit were used for regression analysis, and the values of Akaike information criterion (AIC) and Bayesian information criterion (BIC) of each model were calculated to further determine which model was suitable for the present study. Under the mixed logit model, the AIC and BIC values of the regression analysis were 1825.801 and 1925.380, respectively. Under the conditional logit model, AIC and BIC values are 2083.552 and 2139.564, respectively. So mixed logit model was applied for analyses in the present study.

The mixed logit estimates are presented in Table 4. As can be seen, all the attributes (“monthly fee,” “distance,” “location,” “medical facility,” “environment” and “nursing staff”) and levels were statistically significant, indicating heterogeneity among preferences for nursing homes. The coefficients are in accordance with expectation and common sense. The absolute values of coefficients, show the extent of their effects. Professional nursing staff (*β* = 2.453, *P* < 0.001) have the biggest effect on the choices, followed by medical facility (*β* = 1.500, *P* < 0.001). Compared with monthly fee more than CNY4000, respondents preferred less than CNY2000 (*β* = 1.345, *P* < 0.001) and CNY 2001 to 4000 (*β* = 1.053, *P* < 0.001). Short distance of nursing homes (*β* = 0.899 and 0.637, respectively) is more attractive than those with good environment (*β* = 0.350, *P* < 0.01 ) and located in downtown (*β* = 0.251, *P* < 0.05) (Table 4).

**Table 4:** Mixed-logit model estimates (*n*=233)

Attributes and levels	β	SE	95% CI
Monthly fee (ref: > CNY4000)			
≤ 2000	1.345***	0.233	(0.888, 1.802)
2001–4000	1.053***	0.177	(0.707, 1.399)
Distance (ref: >60 min)			
≤ 30 min	0.899***	0.203	(0.502, 1.297)
31–60 min	0.637***	0.172	(0.299, 0.974)
Location(ref: suburb)			
Downtown	0.251*	0.117	(0.021, 0.480)
Medical facility (ref: no)			
Yes	1.500***	0.243	(1.023, 1.977)
Environment (ref: fair)			
Good	0.350**	0.1301	(0.095, 0.605)
Nursing staff (ref: not professional)			
Professional	2.453***	0.315	(1.835, 3.071)
Log likelihood	-896.901		
AIC	1825.801		
BIC	1925.380		
Sample size	233		
Observations	3728		

*β* Beta Coefficient, *SE *Standard Error, *CI *Confidence Interval

*P < 0.05, ** *P* < 0.01, *** *P* < 0.001

### Preference for nursing homes according to gender and age

The DCE results according to gender and different age group are shown in Tables 5 and 6. Compared with the female, the males preferred professional nursing staff (*β* = 2.939 vs. *β* = 2.643, *P* < 0.001), medical facilities (*β* = 1.890 vs. *β =* 1.498, *P* < 0.001), and the environment of the nursing home (*β* = 0.752, *P* < 0.01). The females are more concerned about the distance and location (*β* = 0.359, *P* < 0.05) of the nursing home (Table 5).

**Table 5:** Sub-group mixed-logit analysis: gender

Attributes and levels	Male			Female		
	β	SE	95%CI	β	SE	95%CI
Monthly fee (ref: > CNY4000)						
≤ 2000	1.648***	0.446	(0.773, 2.523)	1.356***	0.332	(0.706, 2.006)
2001–4000	1.081***	0.288	(0.517, 1.645)	1.333***	0.326	(0.694, 1.972)
Distance (ref: >60 min)						
≤ 30 min	0.673	0.348	(-0.010, 1.355)	1.133***	0.280	(0.584, 1.682)
31–60 min	0.085	0.268	(-0.440, 0.610)	1.083***	0.271	(0.552, 1.615)
Location(ref: suburb)						
Downtown	0.189	0.207	(-0.217, 0.595)	0.359*	0.157	(0.051, 0.668)
Medical facility (ref: no)						
Yes	1.890***	0.453	(1.002, 2.779)	1.498***	0.316	(0.879, 2.118)
Environment (ref: fair)						
Good	0.752**	0.231	(0.300, 1.205)	0.077	0.177	(-0.270, 0.424)
Nursing staff (ref: not professional)						
Professional	2.939***	0.616	(1.732, 4.145)	2.643***	0.498	(1.667, 3.619)
Log likelihood	-362.901			-521.062		
Sample size	100			133		
Observations	1600			2128		

*β* Beta Coefficient, *SE *Standard error, *CI *Confidence interval

*P < 0.05, ** *P* < 0.01, *** *P* < 0.001

Different age groups have different preferences for nursing homes. Old people aged 60–69 preferred professional nursing staff (*β* = 3.870, *P* < 0.01) and medical facilities (*β* = 2.607, *P* < 0.05); old people aged 70–70 are more concerned about monthly fee and the distance from home; For those aged 80 years and older, they only preferred professional nursing staff (*β* = 2.657, *P* < 0.05) and medical facilities (*β* = 1.993, *P* < 0.05), this group don’t concern about other attributes such as monthly fee, distance and location (Table 6).

**Table 6:** Sub-group mixed-logit analysis: age

Attributes and levels	60–69			70–79			≥ 80		
	β	SE	95%CI	β	SE	95%CI	β	SE	95%CI
Monthly fee (ref: > CNY4000)									
≤ 2000	1.474*	0.569	(0.358, 2.590)	1.783***	0.400	(0.999, 2.567)	1.964	1.004	(-0.003, 3.931)
2001–4000	1.541**	0.504	(0.553, 2.529)	1.653***	0.0385	(0.899, 2.407)	0.351	0.516	(-0.660, 1.362)
Distance (ref: >60 min)									
≤ 30 min	1.098	0.560	(0.001, 2.196)	0.717**	0.273	(0.181, 1.252)	2.661	1.361	(-0.008, 5.329)
31–60 min	0.530	0.482	(-0.415, 1.474)	1.008**	0.309	(0.402, 1.614)	0.992	0.657	(-0.296, 2.279)
Location(ref: suburb)									
Downtown	0.459	0.368	(-0.263, 1.180)	0.255	0.195	(-0.127, 0.638)	0.317	0.347	(-0.363, 0.998)
Medical facility (ref: no)									
Yes	2.607*	1.169	(0.316, 4.897)	1.625***	0.394	(0.852, 2.398)	1.993*	0.875	(0.278, 3.708)
Environment (ref: fair)									
Good	1.003*	0.458	(0.105, 1.900)	0.131	0.192	(-0.245, 0.507)	0.207	0.383	(-0.544, 0.958)
Nursing staff (ref: not professional)									
Professional	3.870**	1.319	(1.285, 6.456)	2.933***	0.615	(1.728, 4.138)	2.657*	1.045	(0.609, 4.704)
Log likelihood	-328.258			-416.398			-131.940		
Sample size	89			110			34		
Observations	1424			1760			544		

*β* Beta Coefficient, *SE *Standard error, *CI *Confidence interval

*P < 0.05, ** *P* < 0.01, *** *P* < 0.001

## Discussion

By using a population-based sample of individuals aged 60 and above in Nantong and Yangzhou, China, the present study examined the preference for nursing homes based on discrete choice experiment. This study indicated that the attributes of nursing homes, including nursing staff, environment, monthly fee, distance to home, environment and location, significantly influence the old people’ choices. The findings are in accordance with previous studies [6, 9]. The findings can help the government optimize resource allocation so that the provision of nursing homes can be well suited to old people’s varied preferences.

In the present study, respondents paid most attention to professional nursing staff. Nursing staff is the dominant input in the production of nursing home services. In China, more than 10 million care staffs are required, while the actual number of employees is less than 1 million [25]. Most front-line nursing staff are women aged 40–59 years with poor training and low levels of education [26]. A survey conducted in northeast China showed that only 8.6% of the nursing staff had a college degree or above and only 53.5% had work qualification certificates [25]. The shortage of professional nursing staff is a particular problem to ensure quality of care services. The government should use policy guidance, attract more high-quality talent to enter the field of geriatric healthcare, improve vocational qualification certification and training systems, and improve the professional level of the geriatric industry [24, 26, 27]. In addition, the social and financial status of the nursing staff should be improved so as to improve occupational attraction [27, 28].

The second most important attribute of nursing home is” medical facility “. In our study, 76.4% of the respondents had chronic diseases and almost half of them rated their health as less than good.

Old people with poor health have more demands for medical services [3]. Previous studies have shown that Chinese old people have the greatest demand for life care and medical care compared to other care services [29]. Since 2013, the Chinese government has promoted the development strategy of “*yi-yang-jie-he*” as a way to cope with the rapid aging of the society, which was announced in the *Healthy China 2030 Plan* and the *13th Five-year Development Plan*. “*yi-yang-jie-he*”, as a new old-age care model, combines medical care with life care and other relevant old-age care services. In other words, it integrates medical services such as preventive healthcare, diagnosis and treatment, rehabilitation with all aspects of life such as life care, social participation, leisure and entertainment and cultural activities [30]. The focus is on promoting a combination of medical care, nursing care and life care, fostering healthy aging, and facilitating the development of services and industries for the old people [26]. However, the institutions of medical care integrated old age care lack a perfect internal management model, making services less satisfactory. There are also some nursing staff who lack professionalism, generally relying on experience in performing their tasks. In addition, only a limited rage and variety of services were integrated, thereby failing to meet the needs of the old people. In the view of the growing trend of population aging, policy makers should further integrate medical care and old-age care and enable old people to better enjoy their later years, as well as help them achieve a health aging society.

For the majority of old people in China, nursing home is paid by direct out-of-pocket payments.

According to China National Bureau of Statistics, monthly consumption expenditure per capita was about CNY3166 for urban residents and CNY1550 for rural residents respectively in 2021. Given the high cost of nursing home relative to current retirement income levels, many old people can’t afford the cost. In the present study, respondents preferred less than CNY2000 per month. In China, public financing for long-term care facilities including nursing homes includes subsidies to service providers and target groups of old people. On the supply side, these subsidies include financial incentives for the development of nursing homes. On the demand side, the subsidies include allowances, either in cash or vouchers for services to older people, especially those who have low- income [26].

Preference heterogeneity for nursing homes of the respondents was examined across gender and age groups. Female respondents were more concerned about the proximity to their homes, while the males were more concerned about the availability of medical facilities and professional care. Under the background of Chinese traditional culture, many women attach more importance to family. So them are expected to choose nursing homes closer to their own home. For the young elderly, geographical location and distance from home are not the influencing factors, mainly because they can easily take transportation to nursing homes. For the oldest old, they are only concern about the availability of professional nursing staff and medical facilities. This can be interpreted the oldest old have poorer health and have more demand for health service. The oldest old rely more on their children for economic support and daily care, so they pay less attention to other factors, such as monthly fee and distance to nursing homes.

### Strengths and limitations of the study

Though the quantitative study, the present study has contributed to the preference study for nursing homes. Strength included the DCE was used in the study and it was more theoretically reliable on quantifying the importance of attributes than other methods [5]. The findings gained in this study can deepen the understanding of old people’s choice preference for nursing homes in the Chinese contexts. These findings are essential for optimal resource allocation for policy makers. Some limitations should be stated. Firstly, the study was conducted in Jiangsu Province, an economically advanced coastal area. The sample included only two cities in one province. Hence, general conclusions cannot be draw and more regions should be included in future studies to increase generalizability. Secondly, DCE studies can be subject to bias. Participants may choose a scenario which is familiar to their current situation, rather than they truly prefer. Thirdly, the preference of old people for nursing home may change as time goes, the cross-sectional design of this study could not be used to establish causality or temporality. Furthermore, although a qualitative research approach was used to ensure that we included the key attributes, there are still other factors that may influence old people’s choices. For example, the operation models of public nursing homes and private nursing homes are different in China, and future research should consider this attribute.

## Conclusions

The present study provided important insights into the characteristics of nursing homes from the old people perspective. The utility obtained from specific nursing homes differs between the old people. A better understanding of preference for nursing homes might contribute to improving elderly care and optimize resource allocation. Old people’s preference should be taken into account by authorities. They should pay attention to the quality and professional level of nursing homes and improve access to health care resources of nursing homes.

### Supplementary Information

**Supplementary Material 1.**

## Data Availability

The datasets used during the current study are available from the corresponding author on reasonable request.

## Abbreviations

DCE: Discrete choice experiment
AIC: Akaike information criterion
BIC: Bayesian information criterion
