# Supplementary material for: Old people’s preference for nursing homes in East China: a discrete choice experiment
Source: BMC Nurs. 2024 Apr 22;23:254. doi: 10.1186/s12912-024-01907-y (PMC11034096; doi:10.1186/s12912-024-01907-y)
Supplement: Supplementary file 1 — Supplementary Material 1. [file 12912_2024_1907_MOESM1_ESM.zip › interview outline.docx]

**Outline of the Interview**

**Managers of the nursing home**

1. What is the occupancy rate of your nursing home?
2. How much does your nursing home charge?
3. How many caregivers are there in your nursing home? What percentage of them have a medical background?
4. In your opinion, what measures should be taken by the government to promote the development of nursing homes?

**Old people**

1. What's your impression of the nursing home?
2. Have you considered going to a nursing home in the future?
3. What attributes of the nursing homes you prefer most?
4. What problems do you think in nursing homes?
