# Supplementary material for: Old people’s preference for nursing homes in East China: a discrete choice experiment
Source: BMC Nurs. 2024 Apr 22;23:254. doi: 10.1186/s12912-024-01907-y (PMC11034096; doi:10.1186/s12912-024-01907-y)
Supplement: Supplementary file 1 — Supplementary Material 1. [file 12912_2024_1907_MOESM1_ESM.zip › Version B survey on the health status and old-age care.docx]

**Survey on the Health Status and old-age care of old adults (Version B)**

We are members of the research group "Health status and old-age care of old adults" of Nantong University. In order to understand the health status and old-age care of the elderly, we conducted this survey. This survey is anonymous and your information is only used for research analysis. Thank you for your cooperation!

**A. Basic information**

|  | **Questions and choices** |  |
| --- | --- | --- |
| **A01** | **Place of investigation:**  ①Urban ②Rural |  |
| **A02** | **Gender**: ①Male ②Female |  |
| **A03** | **Age** |  |
| **A04** | **What is your marital status?**  ①Married with a spouse ②Widowed ③Divorced ④Unmarried |  |
| **A05** | **What is your education?**  ①Illiterate ②Sishu/home school ③Elementary school ④Middle school ⑤high school/vocational school ⑥Two-/Three-Year College/Associate degree ⑦Bachelor's degree and above |  |
| **A06** | **Are you currently working?**  ① Yes ② No (**skip to A08**) |  |
| **A07** | **What is your current job?**  ① Leaders of the state, enterprises and institutions ② Professional and technical staff  ③ General office staff ④General workers in commerce/service/manufacturing  ⑤ Self-employed, freelancers ⑥ Farmers, herders, fishermen ⑦ Others |  |
| **A08** | **What is your job before you stopped working?**  ① Leaders of the state, enterprises and institutions ② Professional and technical staff  ③ General office staff ④ General workers in commerce/service/manufacturing  ⑤ Self-employed, freelancers ⑥ Farmers, herders, fishermen ⑦ Others |  |
| **A09** | **Do you have a pension?** ① Yes ② No |  |
| **A10** | **What is your current monthly income?**  CNY |  |
| **A11** | **How do you feel about your family's financial situation?**  ①Very good ②Good ③Average ④Not good ⑤Very bad |  |
| **A12** | **How many sons do you have?** |  |
| **A13** | **How many daughters do you have?** |  |
| **A14** | **How often do you see your children?**  ①Almost every day ②At least once a week ③At least once a month ④Once every few months ⑤Almost never see each other |  |
| **A15** | **How often do you contact with your children? (including phone calls, videos, letters, etc.)**  ①Almost every day ②At least once a week ③At least once a month ④Once every few months ⑤Never contact |  |
| **A16** | **How many people (including yourself) live with you? (If living alone, skip to A18)** |  |
| **A17** | **Who are them? (Multiple choice)**  (1) Spouse (2) Unmarried partner (3) Son (4) Daughter (5) Parent (6) Spouse's parent (7) Sibling (8) Son-in-law (9) Daughter-in-law (10) grandchild or his/her spouse (11) Nanny (12) Other |  |
| **A18** | **Are you satisfied with your life?**   1. very satisfied ②satisfied ③fair ④dissatisfied ⑤very dissatisfied |  |

**B. Health status**

| **B01** | **What’s your self-rated health?**  ① Very good ② Good. ③ Average ④ Not good ⑤ Very bad |  |
| --- | --- | --- |
| **B02** | **Are you currently suffering from a chronic disease?** ① Yes ② No (**skip to section C**) |  |
| **B03** | **What chronic conditions do you suffer from? (Multiple answers possible)**  (1) Hypertension (2) Heart disease/coronary heart disease (3) Diabetes mellitus  (4) Cerebrovascular disease (including stroke) (5) Kidney disease (6) Liver disease  (7) Tuberculosis (8) Rheumatoid (9) Cervical/lumbar diseases (10) Arthritis (11) Breast diseases (12) Reproductive system diseases (13) Prostate diseases (14) Urological Diseases  (15) Glaucoma/Cataracts (16) Cancer/Malignant Tumors (17) Alzheimer's Disease  (18) Osteoporosis (19) Chronic bronchitis/other respiratory diseases (20) Neurological diseases (21) Gastroenteritis or other digestive diseases (22) Parkinson's (23) Deafness  (24) Other chronic diseases |  |

**C. Activities of daily living**

|  |  | No difficulty Difficult but can do Need help Can't do it |  |
| --- | --- | --- | --- |
| **C01** | Dress | ① ② ③ ④ |  |
| **C02** | Take a bath | ① ② ③ ④ |  |
| **C03** | Eat | ① ② ③ ④ |  |
| **C04** | Getting into/out of bed | ① ② ③ ④ |  |
| **C05** | Use the toilet | ① ② ③ ④ |  |
| **C06** | Control urination and defecation | ① ② ③ ④ |  |

**D. Preferences for nursing home (version B)**

Assume that there are two nursing homes to choose, and the two nursing homes differ in different level of attributes. Of the nine senarios listed below, please select the nursing home you prefer. When making your choice, it is assumed that all the conditions of nursing home 1 and nursing home 2 are the same except for the six attributes.

**D01 Scenario 1**

| **Attributes** | **Nursing home 1** | **Nursing home 2** |
| --- | --- | --- |
| Monthly fee | CNY2001~4000 | >CNY4000 |
| Distance from home | 31-60 minutes | ≤30 minutes |
| Location | suburb | downtown |
| Medical facility | no | yes |
| Environment | good | fair |
| Nursing staff | not professional | professional |
| Which nursing home would you prefer to choose? | □ | □ |

**D02 Scenario 2**

| **Attributes** | **Nursing home 1** | **Nursing home 2** |
| --- | --- | --- |
| Monthly fee | >CNY4000 | ≤CNY2000 |
| Distance from home | 31-60 minutes | >60 minutes |
| Location | suburb | downtown |
| Medical facility | yes | no |
| Environment | fair | good |
| Nursing staff | professional | not professional |
| Which nursing home would you prefer to choose? | □ | □ |

**D03 Scenario 3**

| **Attributes** | **Nursing home 1** | **Nursing home 2** |
| --- | --- | --- |
| Monthly fee | >CNY4000 | CNY2001~4000 |
| Distance from home | >60 minutes | ≤30 minutes. |
| Location | downtown | suburb |
| Medical facility | yes | no |
| Environment | good | fair |
| Nursing staff | professional | not professional |
| Which nursing home would you prefer to choose? | □ | □ |

**D04 Scenario 4**

| **Attributes** | **Nursing home 1** | **Nursing home 2** |
| --- | --- | --- |
| Monthly fee | >CNY4000 | ≤CNY2000 |
| Distance from home | >60 minutes | ≤30 minutes. |
| Location | downtown | suburb |
| Medical facility | yes | no |
| Environment | fair | good |
| Nursing staff | not professional | professional |
| Which nursing home would you prefer to choose? | □ | □ |

**D05 Scenario 5**

| **Attributes** | **Nursing home 1** | **Nursing home2** |
| --- | --- | --- |
| Monthly fee | CNY2001~4000 | ≤CNY2000 |
| Distance from home | ≤30 minutes. | >60 minutes |
| Location | downtown | suburb |
| Medical facility | no | yes |
| Environment | good | fair |
| Nursing staff | professional | not professional |
| Which nursing home would you prefer to choose? | □ | □ |

**D06 Scenario 6**

| **Attributes** | **Nursing home 1** | **Nursing home 2** |
| --- | --- | --- |
| Monthly fee | >CNY4000 | ≤CNY2000 |
| Distance from home | >60 minutes | 31-60 minutes |
| Location | downtown | suburb |
| Medical facility | no | yes |
| Environment | good | fair |
| Nursing staff | not professional | professional |
| Which nursing home would you prefer to choose? | □ | □ |

**D07 Scenario 7**

| **Attributes** | **Nursing home 1** | **Nursing home 2** |
| --- | --- | --- |
| Monthly fee | CNY2001~4000 | >CNY4000 |
| Distance from home | >60 minutes | 31-60 minutes |
| Location | suburb | downtown |
| Medical facility | yes | no |
| Environment | good | fair |
| Nursing staff | professional | not professional |
| Which nursing home would you prefer to choose? | □ | □ |

**D08 Scenario 8**

| **Attributes** | **Nursing home 1** | **Nursing home 2** |
| --- | --- | --- |
| Monthly fee | >CNY4000 | ≤CNY2000 |
| Distance from home | 31-60 minutes. | ≤30 minutes |
| Location | suburb | downtown |
| Medical facility | no | yes |
| Environment | fair | good |
| Nursing staff | not professional | professional |
| Which nursing home would you prefer to choose? | □ | □ |

**D09 Scenario 9**

| **Attributes** | **Nursing home 1** | **Nursing home 2** |
| --- | --- | --- |
| Monthly fee | >CNY4000 | CNY2001~4000 |
| 访谈提纲  Distance from home | >60 minutes | ≤30 minutes. |
| Location | downtown | suburb |
| Medical facility | yes | no |
| Environment | good | fair |
| Nursing staff | professional | not professional |
| Which nursing home would you prefer to choose? | □ | □ |
